# Physical Properties of Composite Films from Tilapia Skin Collagen with Pachyrhizus Starch and Rambutan Peel Phenolics

**DOI:** 10.3390/md17120662

**Published:** 2019-11-25

**Authors:** Yongliang Zhuang, Shiyan Ruan, Hanghang Yao, Yun Sun

**Affiliations:** Faculty of Agriculture and Food, Kunming University of Science and Technology, No. 727 South Jingming Road, Kunming 650500, Yunnan, China; ylzhuang@kmust.edu.cn (Y.Z.);

**Keywords:** collagen, starch, phenolics, properties, composite films

## Abstract

Different composite films composed of tilapia skin collagen (TSC) with Pachyrhizus starch (PS) or rambutan peel phenolics (RPP) were prepared, and the physical properties of these films were determined. The effects of PS and RPP on TSC films were investigated, and our results indicated that PS and RPP could improve the physical properties of TSC films. Opacity and film thickness showed an enhanced trend with increasing PS and RPP contents in TSC films, whereas solubility in water, elongation-at-break (EAB), and water vapor permeability (WVP) showed declining trends. TSC film with 10% PS and 0.5% RPP had the highest tensile strength, and the tensile strength dropped drastically when the content of PS and RPP increased. The light transmittances of the films could decrease with the incorporation of PS and RPP. Differential scanning calorimetry (DSC) demonstrated that the addition of PS and RPP improved the thermal stability of TSC films. In addition, X-ray diffraction indicated that the crystallinity of the films decreased and the amorphous structure of the films tended to become more complex with the addition of PS and RPP. As shown by fourier transform infrared spectroscopy (FTIR) analysis, PS and RPP can strongly interact with TSC, resulting in a modification of its structure. Scanning electron microscope (SEM) analysis showed that there was a good compatibility between TSC, PS, and RPP. The results indicated that TSC film incorporated with 10% PS and 0.5% RPP was an effective method for improve the physical properties of the film. TSC–PS–RPP composite films can be used not only in biomedical applications, but also as active food packaging materials.

## 1. Introduction

In recent years, the application of renewable, degradable natural materials on films has attracted increasing attention [1]. Polysaccharides, proteins, and lipids are usually used as matrixes to prepare films. Protein is the best choice because of its good film formation, gas barrier, and mechanical properties compared with others [2]. Collagen, which is the major structural protein in connective tissues, is widely used to prepare these films. Collagen film, which is widely used in various fields such as medical, pharmaceutical, and cosmetics industries, and is also used for food packaging, was successfully commercialized in 1980s [3]. Collagen can be used as a good matrix for film preparation due to its excellent properties, including non-toxicity, biocompatibility, low antigenicity and allergenicity, and biodegradability [4,5]. However, the low viscoelasticity and strong hydrophilicity of collagen film limit its application in some fields. Therefore, collagen film needs to be modified to improve its functional properties.

Starch is becoming a promising alternative for films preparations due to its excellent film-forming and oxygen barrier properties. Starch can effectively enhance the mechanical performance of protein film [6]. Wang et al. modified collagen film with different concentrations of waxy corn starch, common corn starch, and high amylose starch to improve its mechanical properties [7]. Al-Hassan and Norziah found that starch can improve the water vapor permeability, water absorption, mechanical properties, and other general properties of amaranth protein film without affecting the optical properties and thickness of the protein film [8]. Therefore, starch can be used as an excellent modifier for protein films.

Plant phenolics are natural modifiers in the preparation of films. The hydroxyl groups of the phenolics can be cross-linked with various biomacromolecules through hydrogen bonds, improving their mechanical properties. Furthermore, phenolics have the advantages of natural non-toxicity, abundant reserves, and diverse physiological functions. Furthermore, plant phenolics, used as the natural crosslink modifiers of films, also enhance the biological activities of the films, such as antibacterial [9] and antioxidant activities [10].

In our previous research studies, tilapia skin [11], Pachyrhizus [12], and rambutan peel [13] were studied as biomaterials, and tilapia skin collagen (TSC), Pachyrhizus starch (PS), and rambutan peel phenolics (RPP) were obtained. The purpose of the present study was to determine the physical properties of different composite films from TSC, PS, and RPP. The effects of PS and RPP on the physical properties of TSC film were evaluated.

## 2. Results and Discussion

### 2.1. Solubility in Water

**Figure 1:**
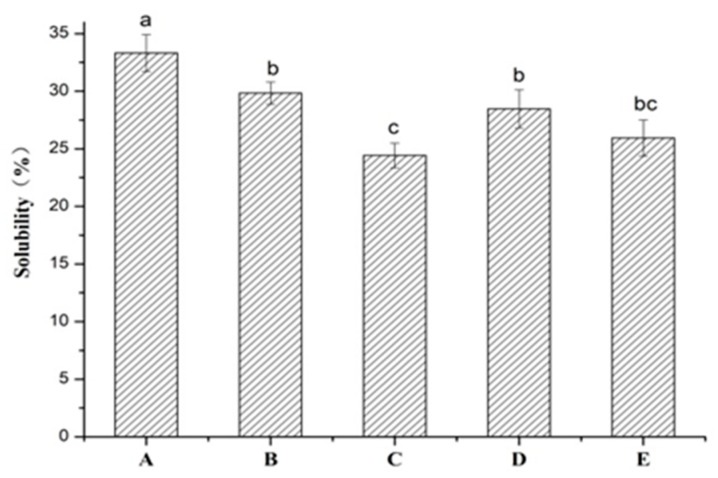
Solubility of the different composite films in water: (**A**) tilapia skin collagen (TSC) film; (**B**) tilapia skin collagen with Pachyrhizus starch (TSC–PS) (10%); (**C**) TSC–PS (50%); (**D**) tilapia skin collagen with Pachyrhizus starch and rambutan peel phenolics (TSC–PS–RPP) (0.5%); (**E**) TSC–PS–RPP (2%). All values are mean ± standard deviation. Different letters indicate significant differences (*p* < 0.05).

The solubility of different composite films in water is displayed in Figure 1. The solubility of TSC–PS (10%) film and TSC–PS (50%) films was 29.84% and 24.42%, respectively, which was significantly lower than that of TSC film (33.31%) (*p* < 0.05). The solubility of TSC–PS films in water decreased with the increase of PS concentrations. The differences in solubility of the TSC–PS films could be attributed to the arrangement and interaction between collagen and starch molecules. Wolf et al. reported that the higher content of starch in collagen films meant that they could form highly cross-linked systems, which could avoid water molecules and dissolve collagen and starch granules by penetrating into the collagen and starch films [14].

As shown in Figure 1, the water resistance ability of the TSC–PS (10%) films was further improved with the different concentrations of RPP, with 28.46% and 25.94% improvements for TSC–PS–RPP (0.5%) and TSC–PS–RPP (2%), respectively. This decrease of the solubility might be related to the network structures of the films. Due to the smaller molecule weights of phenolics, they could penetrate into the interior space between the collagen and starch and cross-link with them through hydrogen bonds to form a more tight and compact structure and prevent the composite films being dissolved [15].

### 2.2. Water Vapor Permeability

Water vapor permeability (WVP) is an important property for evaluating the mechanisms of water transfer and the barrier performance of the films [16]. The main function of the films is usually to avoid moisture transfer between a substance and the surrounding atmosphere, so WVP values should be as low as possible. The lower WVP values of the films show their better performance.

As shown in Table 1, the WVP value of the TSC film was the highest, at 8.89 × 10^−14^ kg·m/(m^2^·s·Pa). The addition of different concentrations of PS and RPP decreased the WVP values of the films. The WVP values of TSC–PS (50%) film and TSC–PS–RPP (2%) film were lower than the TSC film, at 7.83 × 10^−14^ kg·m/(m^2^·s·Pa) and 7.96 × 10^−14^ kg·m/(m^2^·s·Pa), respectively. On the one hand, this may have been due to the cross-linking of hydrogen bonds between TSC and PS, which increased the order and crystallization of TSC–PS films and hindered the free diffusion of water molecules. On the other hand, since starch itself is a macromolecular material with good crystallinity, it can increase the crystallinity of the films and improve their density. In addition, our results showed that the increase of phenolics reduced the WVP values of the composite films to some extent. Since the phenolics contained a large amount of hydroxyl groups on the benzene ring [17], the addition of RPP to TSC–PS film led to the increase of hydrogen bond densities between TSC and PS [18], and made the molecular structure of TSC–PS films denser, thereby enhancing their water vapor barrier properties. It was reported that the WVP value of polyethylene common plastic film is 2.935 × 10^−3^ kg·m/(m^2^·s·Pa) [19]. Obviously, TSC–PS–RPP film had a lower WVP compared with that of the plastic film. The low WVP values of the films will help to reduce or avoid moisture exchange between food and the surrounding environment.

### 2.3. Thickness and Mechanical Properties

Film thickness is a very important parameter, which is directly related to whether the film material works properly. In addition, the mechanical properties and light transmission properties of the film materials are closely related to its thickness. As shown in Table 2, it was observed that the addition of PS and RPP to TSC film caused no significant difference in the thickness of all films (*p* > 0.05).

The mechanical properties of the films are important for their practical applications. The tensile strength and elongation-at-break values of the different composite films are shown in Table 2. It can be seen that tensile strength of the films showed no significant differences (*p* > 0.05), except TSC–PS (50%) film. The significant decrease in tensile strength of TSC–PS (50%) film may have been due to aggregation of residual starch, which destroyed the original uniform network structure between TSC and PS, and reduced the adhesion of the film matrix [20,21].

Compared with the tensile strength, the elongation-at-break values of the films had a similar regularity. The addition of different concentrations of PS and RPP reduced the elongation-at-break of TSC film. Among them, TSC film had the highest elongation-at-break, showing 14.41% (Table 2). The elongation-at-break of TSC–PS (50%) film was the lowest, showing 9.06%. The decrease in elongation-at-break may have been due to the cross-linking reaction between starch, phenolics, and collagen, and this reaction made the structure of the film matrix more stable. The results for tensile strength and elongation-at-break showed that TSC–PS–RPP (0.5%) was optimal.

### 2.4. Fourier Transform Infrared Spectroscopy (FTIR) Spectra

**Figure 2:**
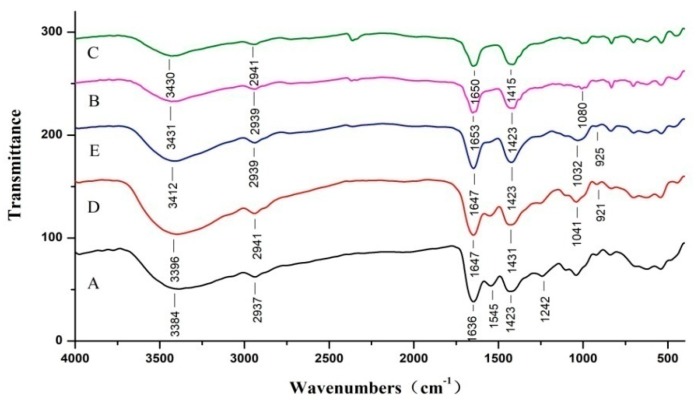
FTIR Spectra of the different composite films: (**A**) TSC film; (**B**) TSC–PS (10%); (**C**) TSC–PS (50%); (**D**) TSC–PS–RPP (0.5%); (**E**) TSC–PS–RPP (2%).

The functional groups and structural changes were detected by FTIR to illustrate the influences of PS and RPP on modifying the structure of TSC film. Figure 2 depicts the FTIR absorption bands of TSC, TSC–PS, and TSC–PS–RPP films. As seen in Figure 2, the absorption frequencies of amide A, amide B, amide I, amide II, and amide III bands of the TSC film were 3384cm^−1^, 2937 cm^−1^, 1636 cm^−1^, 1545 cm^−1^, and 1242cm^−1^, respectively. The amide A band is produced by N-H stretching vibration, the amide B band is produced by the asymmetric stretching vibration of CH_2_, the amide I band is produced by C=O stretching vibration, the amide II band is produced by the bending vibration of CH_2_ and stretching vibration of the C-N bond, and the amide III band is produced by the swinging vibration of CH_2_ [22,23]. After blending with starch, the characteristic absorption peaks of TSC–PS (10%) and TSC–PS (50%) films changed obviously. The bands of TSC–PS (10%) and TSC–PS (50%) films in the amide A and amide I regions shifted to a higher wave number compared to TSC film, which may be relevant for alterations in interactions of TSC and PS molecules, such as H-bonds [24]. The results were consistent with the study of Wang et al. [25].

After modification of RPP, the amide A and B bands of TSC–PS–RPP films exhibited similar infrared absorption characteristics to TSC–PS (10%) films. No absorption peak shift or new absorption peaks were found. This indicated that the addition of RPP had little effect on the infrared absorption of the amide A and B bands of the TSC film, and the polyphenol molecules were evenly distributed between collagen and starch molecules. In the low-wave-number fingerprint region (1330–400 cm^−1^), the infrared spectra of the different composite films still showed a high similarity. With the increase of RPP concentrations, the absorption frequencies shifted to 1041cm^−1^ and 1032 cm^−1^, respectively. The peaks may have been generated by the stretching vibration of ether bonds on the pyran sugar ring [26]. These changes showed that there was an interaction between RPP and TSC–PS films.

### 2.5. Opacity and Light Transmittance

**Figure 3:**
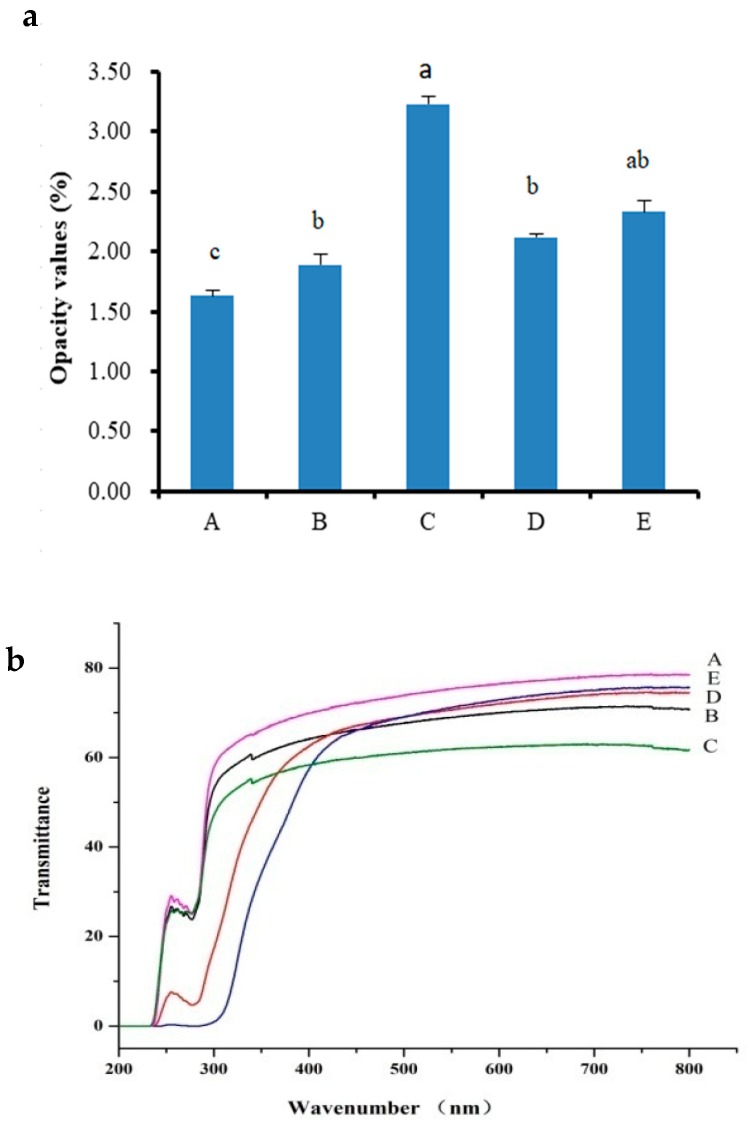
Opacity value (**a**) and light transmittance (**b**) of the different composite films: (**A**) TSC film; (**B**) TSC–PS (10%); (**C**) TSC–PS (50%); (**D**) TSC–PS–RPP (0.5%); (**E**) TSC–PS–RPP (2%). All values are mean ± standard deviation, and different letters indicate significant differences (*p* < 0.05).

Opacity and light transmittance are critical properties for the film applications, particularly in the use of film to improve product appearance [27,28]. Opacity value corresponds to a higher visible light absorbance of a film sample at a given thickness, and light transmittance indicates the ability of the film to absorb light. Opacity values of the different composite films are shown in Figure 3a. The opacity values of TSC–PS films increased significantly with the increasing additive amount of PS (*p* < 0.05). Moreover, the addition of RPP increased the opacity values of TSC–PS films, and the opacity values of TSC–PS, TSC–PS–RPP (0.5%), and TSC–PS–RPP (2%) films were 1.89%, 2.12%, and 2.33%, respectively. Opacity values, associated with the appearance and color of the films [29], play a key role in food coating or food packaging [30]. The increase of the opacity values of the TSC–PS–RPP films indicated that the RPP could achieve a certain degree of cross-linking with TSC and PS and reduce the permeability path and permeability of the films.

Light transmittance values of the different composite films at 200–800 nm wavelengths are shown in Figure 3b. In the range of 200–235 nm, the transmittance of all films was 0, which indicated that the ultraviolet light was completely absorbed by these films. This phenomenon may be caused by the destruction of the collagen triple helix structure during the preparation of the collagen film, which led to the exposure of the chromophore group hidden inside the collagen [31]. In the ultraviolet region (235–400 nm), the transmittance curves of the different composite films increased in a “stepwise” manner. As seen from Figure 3, all films had good light transmittance in the visible light range (400–760 nm). Among them, the transparency of TSC film was the best, at about 70%. With the increase of starch concentrations in the film blends, the transparency of the films decreased. This result may have been due to the hydrogen bond cross-linking between starch and collagen, which increased the density of the films, reduced the permeability path and the amount of penetration of the incident light, and thus decreased the light transmittance.

After modification of RPP, TSC–PS–RPP films had better light transmittance than TSC–PS films. When the concentration of RPP was 2%, the transmittance was the highest, at about 70%. In the ultraviolet region with a wavelength below 450 nm, the transmittance of the film gradually decreased with the increase of the RPP content. The decrease in the transmittance of the films was closely related to the structure of the phenolics. Previous studies have shown that the π electrons on the conjugated double bond of the aromatic compound can absorb light of a certain wavelength and produce strong ultraviolet absorption in the near ultraviolet region. When RPP was combined with TSC and PS, the film had strong ultraviolet light absorption ability. Therefore, the transmittance of the TSC–PS–RPP film in the ultraviolet region was lowered. The lower UV transmittance indicated that the films were more suitable for use as an ultraviolet photoresistance material [32].

### 2.6. Thermal Stability

**Figure 4:**
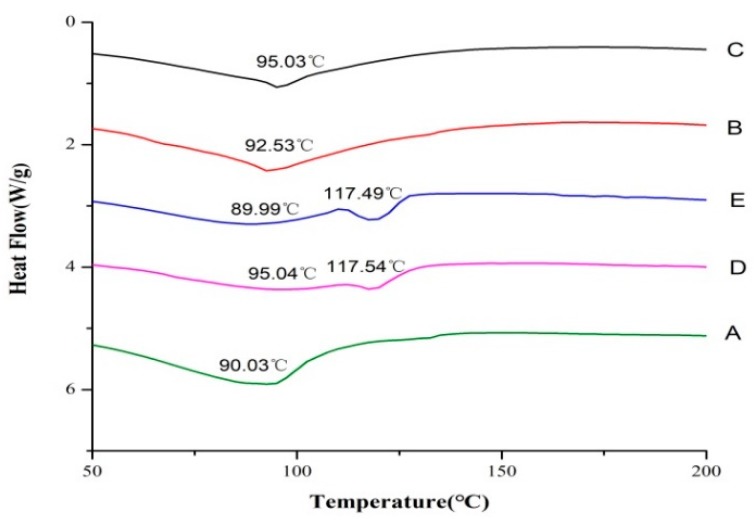
Differential scanning calorimetry (DSC) thermogram of the different composite films: (**A**) TSC film; (**B**) TSC–PS (10%); (**C**) TSC–PS (50%); (**D**) TSC–PS–RPP (0.5%); (**E**) TSC–PS–RPP (2%).

Differential scanning calorimetry (DSC) is a thermal analysis technique that can measure the change in energy with temperature between a sample and a reference [33]. It is often used to study the characteristics of a sample, such as its purity, thermal denaturation temperature (Td), reaction heat, phase diagram, and reaction rate. Phase changes and other thermal processes can cause a difference in heat flow, and provide information about the phase transition of a sample. Figure 4 shows the DSC thermogram of the different composite films in temperature range of 50–200 °C. As shown in Figure 4, both TSC and TSC–PS films had only one endothermic peak (T_1_) in the range of 50–200 °C, which was the Td of these films. Td was caused by the destruction of intramolecular or intermolecular hydrogen bonds and van der Waals forces in collagen [34]. Among them, TSC film had the lowest Td of 90.03 °C. After modification by starch, the Td values of TSC–PS films were improved compared with that of TSC film, showing 92.53 °C and 95.0 °C, respectively. The change in the Td of the films was also related to the cross-linking of hydrogen bonds between TSC and PS. With the higher degree of cross-linking, greater energy was required to destroy the structure. Furthermore, with the addition of RPP to TSC–PS films, the T1 values of TSC–PS–RPP films changed, and a new absorption peak (T_2_) was produced at about 117 °C. Compared with TSC–PS (10%) film, the Td of T1 of TSC–PS–RPP (0.5%) film increased to 95.04 °C, which may have been due to the further cross-linking of the hydroxyl groups of RPP with TSC–PS films.

### 2.7. X-ray Diffraction

**Figure 5:**
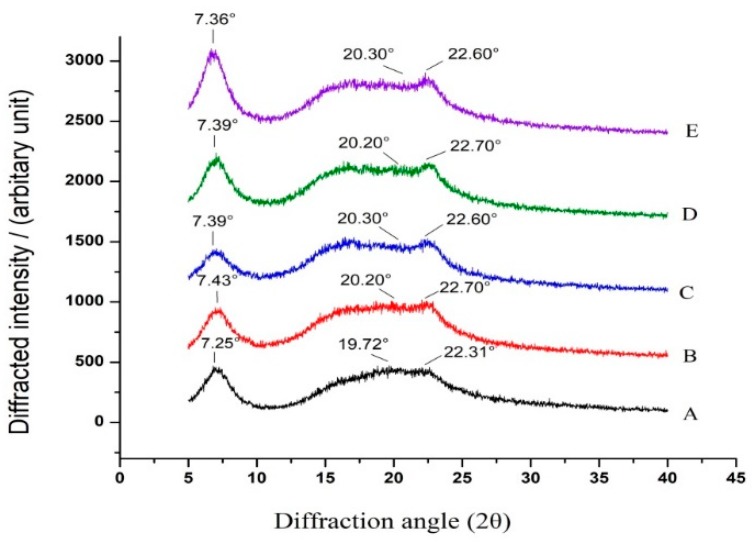
X-ray diffraction patterns of the different composite films: (**A**) TSC film; (**B**) TSC–PS (10%); (**C**) TSC–PS (50%); (**D**) TSC–PS–RPP (0.5%); (**E**) TSC–PS–RPP (2%).

X-ray diffraction (XRD) is a method used to study the crystal structure and atomic arrangement of a sample [35]. Figure 5 shows the XRD spectra of the different composite films. It can be seen that there were three peaks for TSC film in the range of 5–40 °C, and the diffraction angles were 7.25°, 19.72°, and 22.31°, respectively. The first peak was sharp and strong, which was produced by the relatively regular part of the internal structure of collagen. The second peak was short and wide, which was produced by the amorphous part of the TSC film. After blending with starch, the X-ray diffraction patterns of TSC–PS films remained basically unchanged, showing a similar trend. With the increase of PS concentrations, the diffraction intensities of peak 1 gradually decreased, indicating that the crystallinity of the TSC–PS films decreased. This may have been due to the increase of the molecular weight of collagen by the binding of starch molecules to collagen.

As seen in Figure 4, the positions of peak 1 of TSC–PS–RPP films showed no change compared with TSC–PS films, but the diffraction intensities were strengthened, indicating that the crystallinity of TSC–PS–RPP was increased. The diffraction pattern of the amorphous region of the TSC–PS (10%) film was relatively smooth, and the continuity was good. After adding RPP, the diffraction pattern of TSC–PS–RPP films in the range of 15° to 25° became intricate, and the diffraction intensity and diffraction angle were difficult to distinguish. These observations revealed the fact that the cross-linking effect of RPP changed the arrangement and orientation of TSC and PS, and the amorphous region structures of TSC–PS–RPP films tended to be complex [36].

### 2.8. SEM

**Figure 6:**
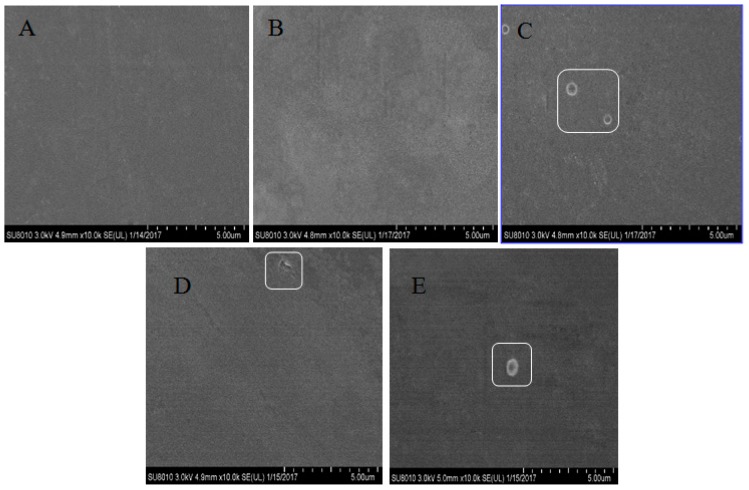
SEM images of the different composite films: (**A**) TSC film; (**B**) TSC–PS (10%); (**C**) TSC–PS (50%); (**D**) TSC–PS–RPP (0.5%); (**E**) TSC–PS–RPP (2%). The enclosed parts represent the residue on the surface of the film.

The surface morphologies of TSC, TSC–PS, and TSC–PS–RPP films were observed using SEM (Figure 6). The TSC film exhibited a relatively smooth surface without any wrinkles. It was clearly observed in the TSC–PS (50%) film that three circular white circles appeared on the surface. This may have been due to the aggregation of starch molecules that were not completely cross-linked, indicating that a small amount of starch may not have been combined with collagen in the films [37]. The morphology results for TSC–PS are in agreement with the previous study [7].

In the TSC–PS–RPP film (Figure 6), the texture of the film had a great similarity to that before modification, keeping its smooth surface, uniform distribution, and good continuity. The addition of RPP did not cause the degradation of the film quality and the matrix was basically a homogeneous system, but the surface morphology of the film was not perfect. From the figure, we can see that there were some white circles on the surface of TSC–PS (50%) and TSC–PS–RPP films, which may be attributed to uncompleted RPP crystallization and ungelatinized starch.

## 3. Materials and Methods

### 3.1. Materials

TSC and RPP were obtained using our previous methods [11,13]. PS was prepared as in our previous study, with some modification [12]. In brief, the Pachyrhizus was cut into lumps and squeezed in a juicer. The resulting Pachyrhizus slurry was then added to distilled water and stirred continuously for 30 h for the starch extraction. After extraction, the slurry was filtered against white gauze (mesh number: 80) and the supernatant containing PS was collected and lyophilized. The timing of glacial acetic acid, glycerol, sodium chloride, ethanol, etc. was purchased from Damao Chemical Reagent Factory (Tianjin, China), all of which were of analytical grade.

### 3.2. Preparation of the Composite Film

First, 2 g TSC was added to 60 mL of acetic acid (0.1 M) and heated at 50 °C or 5 min to obtain TSC solution. For the preparation process of the films, the TSC solution with 30 wt% of glycerol (on the basis of the dry TSC) added as plasticizer was mixed with PS at concentrations of 10 wt% and 50 wt% (TSC film as the control) and named TSC–PS (10%) and TSC–PS (50%), respectively. At last, RPP was added into the TSC–PS (10%) solution at concentrations of 0.5% and 2%, and these were named TSC–PS–RPP (0.5%) and TSC–PS–RPP (2%), respectively. All groups were stirred at 45 °C for 30 min to obtain a uniform mixed solution and then sonicated to remove air bubbles. Then, the dispersions were quickly cast onto a glass plate and put in a ventilation oven at 35 °C for 4 h. The obtained films were stored in desiccators at 50% relative humidity (RH) and 25 °C for 2 days for further analysis.

### 3.3. Film Solubility in Water

The solubility in water of the films was estimated according to a previously published report [38]. Film samples were first immersed in distilled water at room temperature for 1 day and the undissolved portions were filtered out. After drying in a laboratory oven at 25 °C, the undissolved film was weighed. The film solubility (%) was calculated using Equation (1):

(1) $$S=\frac{W_{b}-W_{a}}{W_{b}}\times 100\%$$

where S is the film solubility in water (%); W_b_ is the initial weight of the film sample (g); W_a_ is the weight of the dry sample after being immersed.

### 3.4. WVP

The WVP of the films was determined by the standard method according to American society for testing and materials (ASTM) E96-80, with slight modifications [39]. The opening side of the weighing bottles containing silica gel (0% RH) was sealed with the films and the bottles were transferred into a desiccator with a saturated sodium chloride solution at the bottom (75% RH) and weighted every 1 h. The WVP of the films was calculated according to the following equation:

(2) $$\mathrm{WVP}=\frac{\mathrm{WVTR}\times X}{\Delta P}$$

(3) $$\mathrm{WVTR}=\frac{\Delta m}{\Delta t\times S}$$

where WVP is the water vapor permeability coefficient (g·m/(m^2^·s·Pa)); WVTR is the water vapor transmission rate (g·m^−2^·s^−1^); X is the thickness of the films (m); ΔP is the water vapor pressure difference across the films (Pa); Δm is the weight change of the weighing bottle (g); Δt is the time of gain (s); S is the exposed area of the film (m^2^).

### 3.5. Film Thickness

The thickness of all films was determined using a micrometer (Mitutoyo, No. 293-766, Tokyo, Japan,). Five random measurements of every sample were taken to obtain accurate average.

### 3.6. Opacity

The opacity of the films was evaluated by examining light absorption at the 600 nm wavelength with a UV-visible spectrophotometer (model TU-1901, General analysis, Beijing, China) according to the previous method, with some modifications [40]. The film samples were cut into strips (1 × 4 cm) and placed to one side of the cuvette with the air reference. The opacity value of each film was calculated using the following equation:

O = A_600_/X (4)

where A_600_ is the absorbance at 600 nm and X is the thickness of film (mm).

### 3.7. Tensile Strength and Elongation-at-Break

Tensile strength and elongation-at-break were tested using a CMT4104 (Shanghai, China) electronic universal testing machine [23]. The mechanical properties of the films included tensile strength and elongation-at-break. Film samples were cut into 20 cm × 50 cm pieces according to the size of the mold and fixed for measurement. The initial grip separation and cross-head speed were set at 30 mm and 60 mm/min, respectively.

### 3.8. FTIR

According to a previously published method [41], infrared characteristics of the films were analyzed by a 5700 type Fourier infrared spectrometer (Perkin Elmer, Waltham, MA, USA). Films were cut into suitable sizes and directly placed in the reading area. The FTIR spectra of the film samples were recorded from 4000 to 475 cm^−1^ at a resolution of 4 cm^−1^.

### 3.9. Light Transmittance

The light barrier properties of the films against the ultraviolet (UV) and visible light were determined at selected wavelengths between 200 nm and 800 nm using a spectrophotometer (TU-1901, Beijing, China), according to the method described by Hosseini et al. [42]. Film samples with portions of 10 × 40 mm were placed in the sample chamber of the photometer. The light transmittance of the films was expressed in percentage for each wavelength compared with light that passed through air.

### 3.10. DSC

The thermal properties of collagen films were characterized by a DSC-60plus (Shimadzu, Kyoto Japan), according to a previously published method with a slight modification [43]. Prior to measurement, the film was dehydrated in a desiccator equipped with blue silica gel for 2 weeks, 3 mg of samples were weighed accurately into aluminum pans and sealed, and then scanned from 25 °C to 90 °C with a heating rate of 10 °C/min. An empty aluminum pan was used as a control. The maximum denaturation temperature was recorded as the temperature of each endothermic peak.

### 3.11. XRD

The crystallinity index of the films was analyzed with a X-ray diffractometer (D8 Advance, Bruker, Germany). The films were fixed in a circular clamp on the instrument and scanned from 5° to 40° at a speed of 4°/min. The current and voltage were 40 mA and 40 kV, respectively.

### 3.12. SEM

The films were fixed on the sample holder, vacuum-coated with gold, and sent to the sample chamber. Micrographs of films were taken at magnifications of × 10000 to identify changes of PS and RPP on the surface of TSC films. The surface morphology of the film samples was analyzed by SEM (SU-8100, Hitachi Company, Tokyo, Japan), using a voltage of 20 kV.

### 3.13. Statistical Analysis

The results were performed in triplicate and analyzed using the statistical program for social sciences SPSS 18.0 statistics package program. All data analyses were presented as means ± standard deviation and a value of *p* < 0.05 was considered statistically significant.

## 4. Conclusions

In this study, TSC films incorporating PS and RPP as modifiers were successfully prepared. At the optimum content level, the addition of PS and RPP significantly improved the mechanical properties.

The tensile strength of TSC film increased from 45.33 MPa to 50.97 MPa with the addition of 10% PS and 0.5% RPP. The WVP and light transmittance values of the composite films were also improved as a result of PS and RPP addition. DSC demonstrated that the addition of 10% of PS and 0.5% of RPP could improve the thermal stability of TSC film. The addition of PS and RPP into TSC film resulted in intermolecular interaction, which was confirmed by FTIR spectra and X-ray diffraction results. Furthermore, SEM showed that TSC–PS (10%) and TSC–PS (10%)–RPP (0.5%) films had good compatibility, and the surface of the composite film was smooth, uniform, and dense. In conclusion, the incorporation PS and RPP into TSC film could be used for composite films in the medical, cosmetics, and food industries.

## Figures and Tables

**Table 1:** The water vapor permeability of the different composite films: (A) TSC film; (B) TSC–PS (10%); (C) TSC–PS (50%); (D) TSC–PS–RPP (0.5%); (E) TSC–PS–RPP (2%).

Sample	WVP × 10^−14^ (kg·m/(m^2^·s·Pa))
A	8.89 ± 0.17 ^a^
B	8.41 ± 0.20 ^b^
C	7.83 ± 0.14 ^c^
D	8.21 ± 0.18 ^b^
E	7.96 ± 0.12 ^b,c^

Different letters indicate significant differences (*p* < 0.05).

**Table 2:** Film thickness, tensile strength, and elongation-at-break values of the different composite films: (A) TSC film; (B) TSC–PS (10%); (C) TSC–PS (50%); (D) TSC–PS–RPP (0.5%); (E) TSC–PS–RPP (2%).

Sample	Film Thickness (μm)	Tensile Strength (MPa)	Elongation-at-Break (%)
A	55.33 ± 1.53 ^a^	45.33 ± 1.70 ^a^	14.41 ± 0.77 ^a^
B	53.33 ± 0.58 ^b^	44.05 ± 1.65 ^a^	11.30 ± 0.89 ^b^
C	53.67 ± 1.15 ^b^	30.21 ± 1.22 ^b^	9.06 ± 0.53 ^c^
D	49.00 ± 0.00 ^c^	50.97 ± 2.20 ^a^	12.62 ± 1.01 ^ab^
E	53.67 ± 1.15 ^b^	46.48 ± 0.66 ^a^	11.72 ± 0.59 ^b^

All values are mean ± standard deviation, and different letters in the same column indicate significant differences (*p* < 0.05).
